# Comparison of Two 3D-Printed Indirect Bonding (IDB) Tray Design Versions and Their Influence on the Transfer Accuracy

**DOI:** 10.3390/jcm11051295

**Published:** 2022-02-26

**Authors:** Julius von Glasenapp, Eva Hofmann, Julia Süpple, Paul-Georg Jost-Brinkmann, Petra Julia Koch

**Affiliations:** Department of Orthodontics and Dentofacial Orthopedics, Charité Center for Oral Health Sciences CC3, Charité—Universitätsmedizin Berlin, Aßmannshauser Straße 4-6, 14197 Berlin, Germany; julius.von-glasenapp@charite.de (J.v.G.); eva.hofmann@charite.de (E.H.); julia.suepple@charite.de (J.S.); paul-g.jost-brinkmann@charite.de (P.-G.J.-B.)

**Keywords:** indirect bonding, transfer accuracy, transfer tray, tray design, CAD/CAM, 3D printing

## Abstract

Objective: This study aims to investigate the transfer accuracy of two different design versions for 3D-printed indirect bonding (IDB) trays. Materials and Methods: Digital plaster models of 27 patients virtually received vestibular attachments on every tooth using OnyxCeph³™ (Image Instruments, Chemnitz, Germany). Based on these simulated bracket and tube positions, two versions of transfer trays were designed for each dental arch and patient, which differed in the mechanism of bracket retention: Variant one (V1) had arm-like structures protruding from the tray base and reaching into the horizontal and vertical bracket slots, and variant two (V2) had a pocket-shaped design enclosing the brackets from three sides. Both tray designs were 3D-printed with the same digital light processing (DLP) printer using a flexible resin-based material (IMPRIMO^®^ LC IBT/Asiga MAX™, SCHEU-DENTAL, Iserlohn, Germany). Brackets and tubes (discovery^®^ smart/pearl, Ortho-Cast M-Series, Dentaurum, Ispringen, Germany) were inserted into the respective retention mechanism of the trays and IDB was performed on corresponding plaster models. An intraoral scan (TRIOS^®^ 3W, 3Shape, Copenhagen, Denmark) was performed to capture the actual attachment positions and compared to the virtually planned positions with Geomagic^©^ Control (3D Systems Inc., Rock Hill, SC, USA) using a scripted calculation tool, which superimposed the respective tooth surfaces. The resulting attachment deviations were determined in three linear (mesiodistal, vertical and orovestibular) and three angular (torque, rotation and tip) directions and analyzed with a descriptive statistical analysis. A comparison between the two IDB tray designs was conducted using a mixed model analysis (IBM, SPSS^®^ Statistics 27, Armonk, NY, USA). Results: Both design versions of the 3D-printed IDB trays did not differ significantly in their transfer accuracy (*p* > 0.05). In total, 98% (V1) and 98.5% (V2) of the linear deviations were within the clinically acceptable range of ±0.2 mm. For the angular deviations, 84.9% (V1) and 86.8% (V2) were within the range of ±1°. With V1, most deviations occurred in the mesiodistal direction (3.3%) and in rotation (18%). With V2, most deviations occurred in the vertical direction (3.8%) and in palatinal and lingual crown torque (16.3%). Conclusions: The transfer accuracies of the investigated design versions for 3D-printed IDB trays show good and comparable results albeit their different retention mechanisms for the attachments and are, therefore, both suitable for clinical practice.

## 1. Introduction

Indirect bonding (IDB) describes a procedure in orthodontics in which attachments are bonded to patients’ teeth with the help of a transfer device. It was initially described in 1972 by Silverman et al. [1] as a proposed solution to the high bracket failure rate due to a long setting time of the then-utilized cement. The approach of using an IDB tray to transfer the positions of brackets from a plaster model to patients’ teeth was innovative at that time. Since then, a variety of designs and materials has been tested for this purpose, of which IDB trays composed of polyvinylsiloxane (PVS) proved to provide a clinically sufficient transfer accuracy and are, nowadays, considered as the benchmark [2,3,4].

In general, the IDB method has multiple advantages compared to the conventional approach of direct bonding. Some authors claim it is more accurate [5], offers shorter clinical chair time [6], provides greater comfort for the patient and allows for easier and more precise adjustments when placing the brackets and tubes in an overcorrected position [7,8]. However, studies also conclude that the overall time spent on this indirect approach is longer [9], causes more bracket failures [6,9] and is more expensive [6] than the direct one when adding the extra laboratory expenses and technician salaries. These disadvantages might explain why IDB has not yet been fully integrated into orthodontic practice [10].

In recent years, however, technological and digital progress in the field has promised to facilitate the IDB procedure by saving resources due to virtual bracket and tube positioning and, hence, the direct [11,12,13,14,15,16,17,18,19] or indirect [20] manufacturing of the IDB tray. Hereby, an intraoral scan of the patient’s dental situation is needed as a basis for further processing in an orthodontic treatment planning and simulation software, specifically when virtually placing the attachments on the patient’s teeth and designing an IDB tray based upon this digital set-up.

Given the standard of 3D printers commonly used in an orthodontic practice and the respective IDB resins with their specific material properties, the design of these IDB trays is crucial to successfully transferring brackets and tubes onto the patient’s teeth with a clinically acceptable accuracy. For this, the IDB tray needs to have sufficient occlusal support to ensure a correct vertical position on the tooth surface and a vestibular extension to hold the brackets and tubes with an elaborate retention mechanism in place and, at the same time, allowing an easy removal without the risk of debonding brackets. Nowadays, an individualized tray design that meets all these requirements can be 3D-printed [6,11,13,15,17,18,21,22,23,24,25,26].

We, therefore, aim to investigate the clinical suitability and transfer accuracy of two design versions for 3D-printed IDB trays differing in their bracket and tube retention mechanism.

## 2. Materials and Methods

At first, impressions were taken from 27 patients with a full permanent dentition and with different malocclusions to generate duplicated molds of both dental arches. From each mold, two identical plaster models were fabricated (OCTA-STONE^®^, Heraeus Kulzer, Hanau, Germany), scanned with an intraoral scanner (TRIOS 3W^®^, 3Shape, Copenhagen, Denmark) and imported into the treatment and simulation software OnxyCeph³™ (Image Instruments, Chemnitz, Germany). The software-integrated FA_Bonding module provided virtual attachments from a bracket library and automatically positioned them on the facial-axis point of every tooth surface. If needed, manual adjustments were performed by the same operator in all directions with a maximum distance of 0.2 mm between the bracket base and the tooth surface to ensure an even contact area. In total 1070 brackets (discovery^®^ smart/discovery^®^ pearl brackets Ortho-Cast™ M-Series 0.018 inch (Roth) Dentaurum, Ispringen, Germany) and 420 buccal tubes (Ortho-Cast M-Series tubes, 0.018 inch (Roth), Dentaurum, Ispringen, Germany) were bonded on every present tooth, including the second molars.

**Figure 1:**
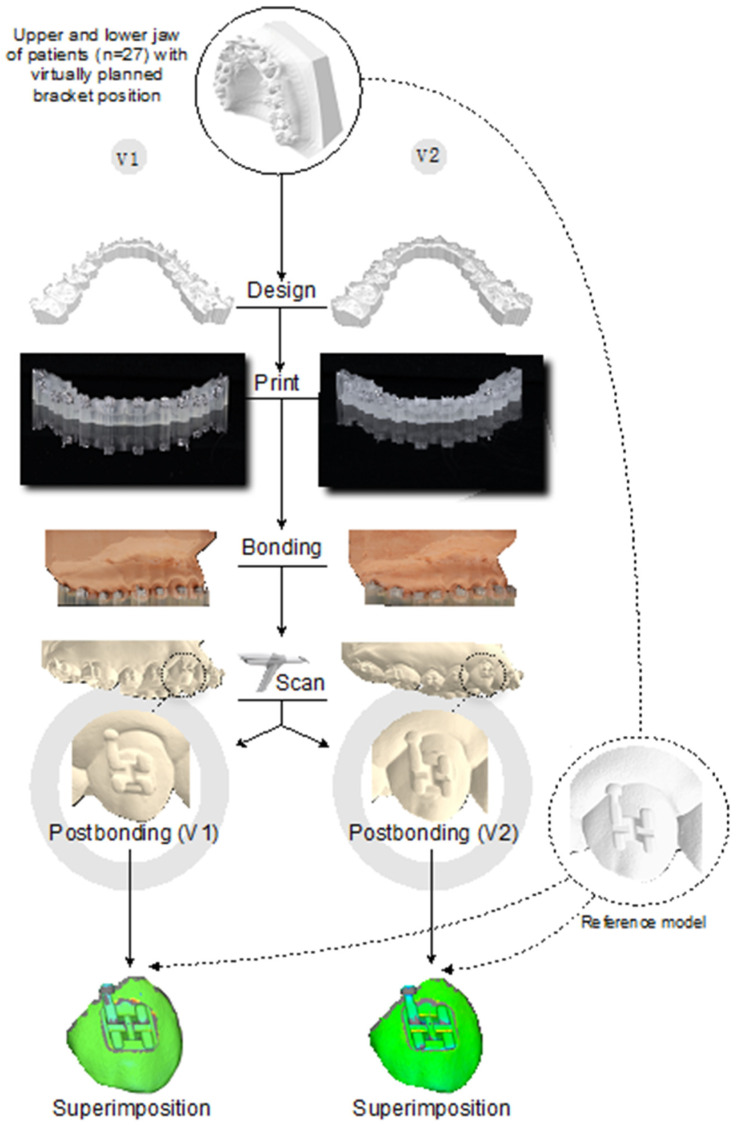
Flow chart.

All models, including their virtually planned attachment positions, were exported and reference models were saved as standard tessellation language (STL-) files for later superimposition when used as reference models (Figure 1).

### 2.1. Virtual Design

Based on these reference models, two IDB tray design versions were designed in the OnyxCeph³™ Bonding Trays 3D module for each dental arch and patient. Several setting parameters in the software offered to modify the shape of the IDB trays to be 3D-printed later and were selected for both design versions, version 1 (V1) and 2 (V2), with respect to the chosen resin and its elastic properties. Each design was finally pretested to ensure that the attachments remained in the holding device during the bonding procedure.

**Figure 2:**
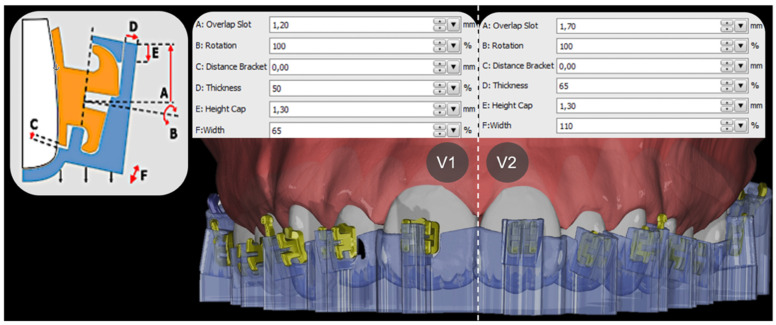
Comparison between version 1 (**V1**) and version 2 (**V2**) tray design and the selected setting parameters. Left: Graphic representation in OnyxCeph³™ of the design possibilities.

V1 and V2 trays mainly differed in their retention mechanism for the selected brackets and tubes and, hence, in the chosen module parameters of the extending tray sleeves, such as occlusogingival bracket overlap, mesiodistal width and orovestibular thickness, as shown in Figure 2.

**Figure 3:**
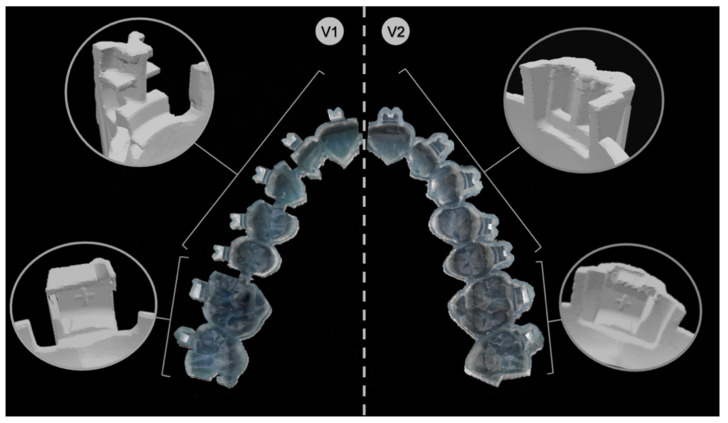
Direct comparison between the design of version 1 (**V1**) and version 2 (**V2**).

Practically, V1 corresponded to all brackets from the second premolar of one quadrant to the second premolar of the adjacent quadrant as arm-like sleeves protruding from the tray base and reaching into the horizontal and vertical bracket slots. Concerning the molars, the set parameters embraced the tubes from the occlusal, vestibular, mesial and gingival sides, leaving out the distal surface. A notch for the hooks prevented distal sliding. Further, V1 trays were segmented into tooth groups with small bridges connecting each section to ensure more flexibility within the tray (Figure 3).

V2 had for all brackets from the second premolar of one quadrant to the second premolar of the adjacent quadrant a pocket-shaped design enclosing the brackets from the occlusal, vestibular, mesial and distal sides, leaving out the gingival surface. Thereby, the fit of the attachments was achieved by clamping. Concerning the molars, the set parameters enclosed the tubes from all sides.

In both IDB tray versions, the tray body covering the occlusal surface of all teeth provided vertical and horizontal support and offered overall stability.

Finally, the IDB trays were virtually generated for all patients in both versions, V1 and V2, and exported as STL-files for 3D printing.

### 2.2. Three-Dimensional Printing

STL-files were imported into the Asiga MAX™ printer software (Asiga Composer, version 1.1.7, Alexandria, Australia). All trays were virtually orientated with the external occlusal surface facing the build platform. To achieve the highest possible accuracy, the maximum resolution of 50 µm slice thickness in Z-axis and 62 µm in X- and Y-axis were selected.

The trays were 3D-printed with digital light processing (DLP) technology, an additive manufacturing process based on a light projector and liquid resin (Asiga MAX™, SCHEU-DENTAL, Iserlohn, Germany). The printer-compatible methacrylate-based material Imprimo^®^ LC IBT (SCHEU-DENTAL, Iserlohn, Germany), which is a flexible (Shore D hardness 40) and transparent light-curing resin material specifically developed for the purpose of 3D printing IDB trays, was used for manufacturing.

After 3D printing, the trays were postprocessed with a jet device using 99% isopropanol, according to manufacturer’s instructions, followed by 10 min in a high-power ultrasonic cleaning bath (IMPRIMO^®^ Clean/IMPRIMO^®^ Cleaning Liquid, SCHEU-DENTAL, Iserlohn, Germany) to remove further resin residues. Finally, the IDB trays were placed into a light-polymerization unit with a nitrogen gas environment (IMPRIMO^®^ Cure, SCHEU-DENTAL, Iserlohn, Germany).

### 2.3. Indirect Bonding

In total, 1070 brackets and 420 tubes (discovery^®^ smart/pearl, Ortho-Cast M-Series, 0.018 inch (Roth) Dentaurum, Ispringen, Germany) were bonded on every vestibular tooth surface, including the second molars. The IDB procedure was performed by the same operator on corresponding plaster models of the 27 in vitro patients fixed in a dental phantom head to simulate an authentic clinical setting. For this, the brackets and tubes were first gently inserted into the IDB trays and their retentive bases were cleaned with cotton pellets drenched in acetone. Then, a thin layer of Transbond™ XT (3M Deutschland GmbH, Neuss, Germany) was evenly applied onto each attachment base until it was fully covered using an application tip. Furthermore, the surface of the plaster models that received the attachments were cleaned with 99% isopropanol and the vestibular surfaces of the teeth were coated with a thin layer of Transbond™ XT Primer (3M Deutschland, Neuss, Germany) on the presumed bracket or tube position.

**Figure 4:**
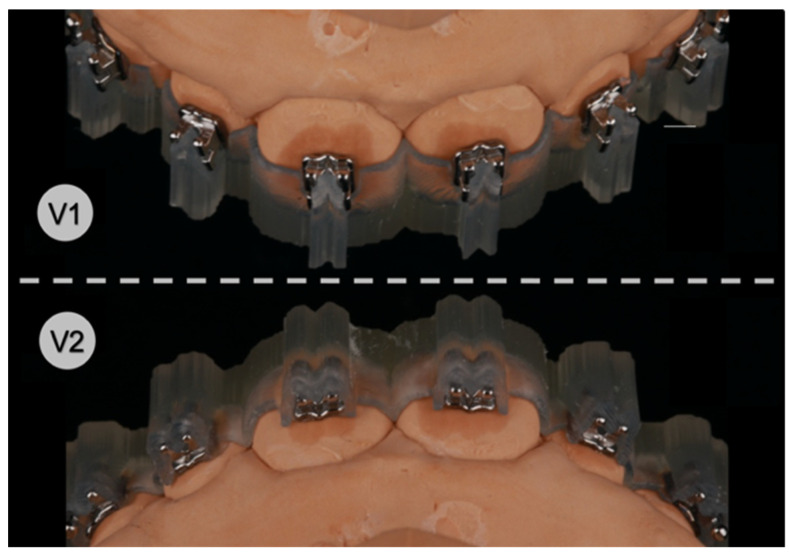
Both transfer tray designs with inserted brackets on stone models, version 1 (**V1**) and version 2 (**V2**).

The IDB trays were then placed onto their corresponding plaster models (Figure 4) and slight finger pressure was applied on the occlusal and vestibular surface to ensure a correct fit. Excess composite leaking lateral from the attachments was removed with a dental probe. Eventually, each bonding unit was light cured for 3 × 4 s in high-power mode (VALO™ Cordless, Ultradent Products Inc., South Jordan, UT, USA; 1400 mW/cm^2^) with the light positioned from all accessible directions, including through the transparent IDB trays into the composite-filled gap between the attachment base and the plaster surface from the smallest possible distance.

After indirect bonding, all IDB trays were carefully removed with a dental scaler by lifting the vestibular extension arms, or the pockets holding the attachments, respectively, and by doing so loosening the respective retention mechanism.

To assess the transfer accuracy of both V1 and V2 IDB trays, another intraoral scan of the final bracket and tube positions was performed using a thin layer of dental scan powder (METAL-POWDER Dry blue, R-dental Dentalerzeugnisse, Hamburg, Germany) beforehand to avoid scan distortions due to reflections of the metallic attachment surfaces.

The obtained digital models with the transferred attachment positions were exported as STL-files for later comparison with the virtually planned attachment positions.

### 2.4. Three-Dimensional Superimposition of Tooth Surfaces

**Figure 5:**
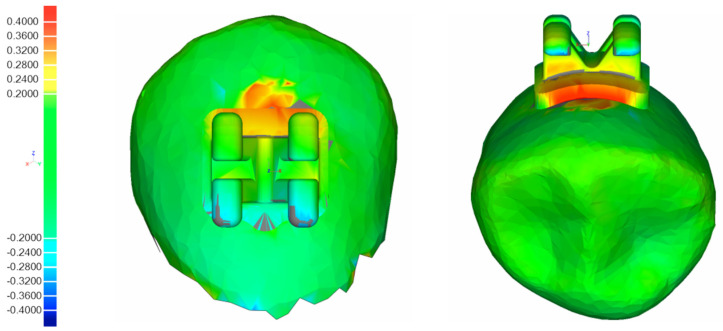
Example of colorimetric best-fit superimposition and bonding inaccuracies.

For every patient and each design version (V1, V2), two STL-files were imported into a 3D inspection software (Geomagic^®^ Control, 3D Systems Inc., Rock Hill, SC, USA), one showing the digital model with the virtually planned bracket and tube positions, the other showing the actual attachment positions after performing IDB. The individual tooth surfaces of the corresponding pre- and postbonding STL-files, which served as reference structures, were separated from the respective dental arches and superimposed using a measurement algorithm specifically scripted for this purpose, first described by Koch et al. [27] conducting an automated and successive best-fit alignment (Figure 5). Since coordinate systems were virtually integrated into every attachment beforehand, the deviations between their respective centers after superimposition were considered equivalent to the difference between the planned and actual attachment positions on the respective vestibular tooth surfaces. Using this procedure, the deviations were quantified in the reference coordinate systems in three linear values along the axes (mesiodistal (X, mm), vertical (Y, mm), orovestibular (Z, mm)) and three angular values around the axes (torque (X, degree), rotation (Y, degree), tip (Z, degree)).

The complete workflow is shown in Figure 1.

### 2.5. Statistical Analysis

A sample size calculation for a paired *t*-test was performed ahead of the investigation (power 80%, α = 2.5%, medium effect size (Cohen’s *d* = 0.667)) and proposed a minimum of 24 patients with full dental arches. The limits for clinical acceptability were set to ±0.2 mm for all three linear directions, according to the *American Board of Orthodontics* [28] and ±1° for all three angular directions, based on previous literature [13,20,21].

To evaluate the deviations from the planned to the real attachment positions, a descriptive analysis (SPSS^®^ Statistics software, version 27.0, Armonk, NY, USA) was conducted for all tooth groups (incisors, canines, premolars and molars) and both IDB tray versions (Table 1). The percentages of brackets and tubes positioned outside the clinically acceptable range are shown in Table 2, considering their directional bias.

A mixed model was chosen for the statistical analysis to compare the two design approaches in terms of their transfer accuracy. The linear and angular values were considered dependent variables, as within a transfer tray a misalignment of one bracket might also affect the position of neighboring brackets. A mixed model takes these random effects into account and was, therefore, chosen as the preferred test (Table 3).

## 3. Results

In total, 1490 brackets and tubes were transferred with both IDB tray design versions (V1, V2). Out of these, 163 had to be excluded from the analysis due to bracket detachment during the bonding procedure ((*n* = 153; V1 (*n* = 66), V2 (*n* = 87)) or an invalid matching process during the superimposition of the tooth surfaces (*n* = 10).

Table 1 presents the mean values and standard deviations of all transferred brackets and tubes in linear and angular dimensions as calculated with absolute numbers. All mean values of both tray design versions were found within the defined clinically acceptable range of ±0.2 mm and ±1°.

For both IDB trays, the linear accuracy was most accurate in the orovestibular direction (V1 = 0.03 mm; V2 = 0.02 mm). The highest linear deviations for V1 were observed in the mesiodistal direction (0.06 mm) and for V2 in the vertical direction (0.07 mm). The highest angular mean values were found with V1 in rotation (0.62°) and with V2 in torque (0.58°).

The directional biases of the transferred brackets and tubes outside the clinical acceptable range of 0.2 mm and 1° are presented in Table 2. With V1, the most frequent linear deviations were observed in mesiodistal direction (3.3%), while 1.8% were placed too far mesially and 1.5% too far distally. With V2, the most frequent linear deviations were observed in vertical direction (3.8%), while 0.9% were placed too far occlusally and 2.9% too far gingivally.

With V1, the most frequent angular deviations were observed in rotation (18%); 9.9% were rotated too far mesially and 8.1% were rotated too far distally. With V2, torque had the most deviations (16.3%); 13.4% of the values were transferred too far in direction of labial crown torque, while only 2.9% were placed too far palatally (palatal crown torque).

The mixed model analysis revealed no significant difference (*p* > 0.05) in the transfer accuracy between the IDB tray design versions V1 and V2. In order to take the accuracy differences within the trays into account, the mixed model also investigated every possible combination of tooth groups and tray designs and concluded no significant difference (Table 3).

## 4. Discussion

In our study, we investigated the clinical suitability and accuracy of two different design versions for 3D-printed IDB trays (V1 and V2) by transferring 1490 brackets and tubes in 27 in vitro patients with full dental arches, including the second molars. Within the presented workflow in the orthodontic laboratory and practice, both tray versions were suitable for application and offered an acceptable transfer accuracy.

For all linear measurements, 98% of V1 and 98.5% of V2 were located within the clinically acceptable set range of ±0.2 mm. Concerning the angular measurements, 84.9% of V1 and 86.8% of V2 did not exceed ±1°.

Other studies obtained comparable results with 3D-printed IDB trays, ranging from 89% to 100% in the linear and 64.5% to 82% in the angular measurements [18,22,23]. In these cases, however, the accepted clinical acceptability ranged from ±0.5 mm to ±2°.

In general, linear deviations in all dimensions were less frequent and distinctive than angular deviations.

For both IDB tray versions, the highest accuracy in the linear dimension was found in orovestibular direction, similar to the results of other studies [13,20,21]. This could be explained by the occlusal relief of the teeth, which functions by its texture as a stop and, therefore, prevents the displacement of the tray and, respectively, its attachments in all directions, specifically in orovestibular direction. On the other hand, the greatest linear deviations occurred in the vertical dimension, especially with tray V2. Its specific design with the pouch-shaped sleeve holding the attachments from all sides except the gingival direction did not prevent them sufficiently from being displaced in vertical direction, although the tray itself was supported by the occlusal surface. The inner hollows of the tray sleeve, which covered the bracket wings, seemed not to print accurately enough for the bracket wings to click into their foreseen space and grasp the attachments properly, which led to a vertical displacement. Tray design V1 retained the brackets actively and, therefore, showed higher vertical accuracies, as it extended into the vertical and horizontal bracket slots, respectively, gingivally overlapping at the tubes. Even if the differences in the vertical displacement between the mean values of both trays were minor with V1 showing fewer deviations, there also seemed to be an influence on the better results for the torque of V1 compared to V2, which showed a strong directional bias of the deviations towards palatal crown torque (13.4%). As found by Miethke and Melsen [29], the mandibular molars, followed by the premolars, showed the largest variation of labial tooth surface with respect to the curvature. Therefore, brackets that were vertically displaced were likely to show higher inaccuracies in torque on molars and premolars than on incisors and canines. Indeed, the results of our study supported this thesis. In both trays, the molars and premolars generally showed higher deviations of the attachments in torque compared to incisors. However, this was not true for canines.

Independent of the vertical displacement, for both design versions, the canines generally showed the highest mean values for angular deviations among all tooth groups. This finding matched the ones of Jungbauer et al. [17] and Koch et al. [30]. Since this observation was independent of both tray design versions and studies, it was likely linked to the rounded tooth morphology of the canines, which caused a point contact of the bracket base to the labial surface. Therefore, depending on the location of the vertically or vestibularly applied finger pressure in relation to the contact point of the canine’s labial tooth surface and the respective bracket base, the brackets rotated during the IDB procedure, causing the mentioned angular deviations.

Therefore, very fine tray structures in the posterior region that could potentially cause printing inaccuracies, as well as applying high finger pressure during IDB, should be avoided.

We, therefore, evaluated the transfer accuracy of 1327 brackets that were bonded from the central incisors to the second premolars and tubes for the first and the second molars. Integrating the second molars was of special interest for us, as bonding in the posterior region is more challenging in general due to impaired visibility and accessibility. Both investigated IDB trays provided information on the transfer accuracy of posterior teeth, which is crucial when it comes to efficiently bonding all attachments together in one step. In contrast, other authors only investigated the transfer accuracy of their respective IDB trays ranging from the central incisors to the second premolars or maximum to the first molars [6,14,15,18,21,22,23]. Consequently, one may suggest that second molars were bonded in an extra step or session or not at all in these studies, which diminishes the gained efficiency when bonding indirectly in the first place.

Czolgosz et al. [6] found computer-aided indirect bonding to be more time consuming in total and that it produced higher bracket debonding rates, which, ultimately, translates into higher costs compared to the direct approach. An adjusted tray design and 3D printing procedure [31] might, therefore, reduce some disadvantages while still profiting from the advantages of a fully digital workflow [31,32].

Regarding the design of the 3D-printed IDB trays, three concepts have been described in the literature so far.

Kim et al. [13], Son et al. [11] and Yue et al. [15] used segmented transfer jigs for every single tooth, whereas Xue and coworkers [14] created a guiding device for the dental arch that framed the planned bracket positions for later direct positioning, therefore, using a hybrid bonding method. Both approaches required the attachments to be bonded one by one and, therefore, lacked the benefit of indirect bonding of brackets and tubes all at once.

Other studies on 3D-printed IDB trays [17,18,23,24,26] used a third concept of an IDB tray covering the dental arch, sometimes including the molars, with pouch-like shaped sleeves holding the attachments in place. We imitated this approach in our tray design V2 and compared its transfer accuracy to a different design version (V1). V1 had the same expansion over the dental arch, including the second molars as V2, but differed in the retention mechanism holding the attachments.

V1 retained the brackets via the horizontal and the vertical bracket slots and, therefore, offered control in all directions, while not extending beyond the mesial, distal or gingival margin of the brackets. This construction offers some clinical advantages regarding the removal of possible composite surplus, but is also more fragile and, therefore, needs careful handling during the application procedure. In contrast, V2 held the brackets by a surrounding sleeve. Although the IDB tray itself was more stable and the brackets appeared more fixed, this design version seemed to be more prone to printing inaccuracies in the fine inner hollows or diffused layer margins, which, ultimately, led to improperly placed brackets in the intended space of the tray, resulting in deviations from the planned position.

As the elasticity of a chosen resin for 3D-printed trays directly correlates to the success of indirect bonding as published by Jungbauer et al. [17] and is predetermined by the industry (Shore-A/Shore-D hardness), we decided to manipulate the elasticity by choosing two design versions that differed in material thickness and its filigree character in specific areas. Unfortunately, the DLP or SLA printing technology does not allow the combination of different materials to be printed simultaneously and, therefore, the mixture of different properties as they are needed within certain areas in an IDB tray is impossible. However, this principle was successfully applied with trays composed of polyvinyl siloxane first described by Nedwed et al. [33]. A hard occlusal support secured the vertical and torque position of the attachments, whereas a more flexible sleeve on the vestibular surface around the attachments ensured the position in other dimensions and enabled an easy removal without risking immediate bracket debonding.

Our design versions both provided a thick and, therefore, stiff occlusal support. The sleeves of V1 and V2 had the same material thickness but different mesiodistal extensions. This led to an increased flexibility of design V1 that prevented immediate bracket debonding. It was subject of our study to investigate if these different design versions had an influence on the bracket transfer accuracy, which we eventually could not confirm.

A limit to our design choice was that the utilized simulation software OnyxCeph^3^™ did not allow different settings to be applied for all brackets and tubes individually, despite their different geometry and, hence, their different requirements for their retention in the tray. Therefore, we needed to compromise on the design of the sleeves holding both the brackets and tubes, resulting in a low retention force for the tubes with tray design V1 and, consequently, in tubes falling out of their mold during application. Furthermore, dental crowding also presented a challenge when bonding indirectly as Jungbauer et al. [17] had pointed out. We previously concluded that better results could be achieved by improving the software’s tools for the 3D simulation of IDB trays by enabling to separately set parameters for selective tooth groups.

Since all IDB trays show fine structures to some degree, they need to be printed with the highest possible resolution of a common contemporary 3D printer. Apart from this and other technical requirements, Arnold et al. [34] also investigated if the setting parameters influence the precision of printed objects such as the positioning, angulation and structure of the object on the build platform of SLA printers.

In our study, we printed V1 and V2 with identical settings and according to manufacturer’s instructions. The resolution was set at maximum, which was 0.062 mm in X- and Y-axis and a layer thickness of 0.05 mm. As also explained by Süpple et al. [20], our resolution settings resulted in nearly cubic shaped voxels, which only showed neglectable effects on the precision of the surface. However, the need for support structures proved to be an influencing factor. The trays were printed horizontally with the flat oral surface of the occlusal part of the tray facing the build platform. Doing so, we aimed at minimizing structural overhangs to the sleeves holding the attachments, which was visually controlled in the slicer software. This way, no support structures were needed. However, we could not exclude the potential occurrence of the common staircase effect at the layer margins, especially in the inner hollows of the trays. The kind of effect that the automatically applied printer software slicing algorithm might have had, determining the alignment of the layers and automatically deciding how curved margins were approximated, can only be speculated.

Generally, we did not notice any visible printing failures and all 3D-printed IDB trays were suitable for indirect bonding.

As for the choice of the printing technology, we used a common desktop DLP printer for manufacturing the trays in combination with a resin specifically offered for IDB. Most studies on the accuracy of 3D-printed objects in orthodontics investigated dental models, which are used for diagnostic reasons or aligner production. Hazeveld et al. [35] evaluated the accuracy of printed dental models using different printing technologies and concluded, that DLP and PolyJet prints are more precise than SLA, and that they are more appropriate for selected appliances in orthodontics. For printing IDB trays, DLP [18,23] and PolyJet [24] technologies have already been investigated before and showed acceptable results for their transfer accuracy despite their different tray designs. Although PolyJet printers are capable of printing two or more different resins in one printing process, which can meet the different local requirements regarding its elasticity as described earlier, and, therefore, seem very promising for this specific use, they are still very costly in their acquisition and are, nowadays, generally used for customed services.

## 5. Conclusions

Both design versions for 3D-printed indirect bonding trays used in this study were clinically suitable and provided an acceptable accuracy in bracket placement.

The V1 tray design transferred attachments just as well as V2, while offering benefits in the handling and its clinical application.

## Figures and Tables

**Table 1:** Absolute mean values and standard deviations between virtually planned and postbonding positions for version 1 (V1) and version 2 (V2).

**V1 ^b^**							
	***n* ^a^**	**Mesiodistal (mm)**	**Vertical (mm)**	**Orovestibular (mm)**	**Torque (°)**	**Rotation (°)**	**Tip (°)**
Incisors	181	0.06 ± 0.06 ^b^	0.07 ± 0.05	0.02 ± 0.02	0.45 ± 0.39	0.69 ± 0.76	0.58 ± 0.53
Canines	105	0.09 ± 0.08	0.06 ± 0.04	0.03 ± 0.02	0.47 ± 0.39	0.98 ± 0.94	0.66 ± 0.58
Premolars	198	0.06 ± 0.06	0.05 ± 0.05	0.03 ± 0.02	0.59 ± 0.55	0.59 ± 0.55	0.43 ± 0.36
Molars	198	0.05 ± 0.05	0.07 ± 0.07	0.03 ± 0.02	0.56 ± 0.55	0.40 ± 0.40	0.19 ± 0.30
Total	679	0.06 ± 0.06	0.06 ± 0.06	0.03 ± 0.02	0.50 ± 0.45	0.62 ± 0.68	0.44 ± 0.47
**V2 ^b^**							
	***n* ^a^**	**Mesiodistal (mm)**	**Vertical (mm)**	**Orovestibular (mm)**	**Torque (°)**	**Rotation (°)**	**Tip (°)**
Incisors	186	0.04 ± 0.04	0.08 ± 0.06	0.03 ± 0.02	0.54 ± 0.41	0.52 ± 0.48	0.66 ± 0.57
Canines	101	0.05 ± 0.04	0.08 ± 0.06	0.03 ± 0.02	0.68 ± 0.60	0.63 ± 0.47	0.68 ± 0.57
Premolars	194	0.05 ± 0.05	0.07 ± 0.05	0.02 ± 0.02	0.58 ± 0.49	0.45 ± 0.47	0.38 ± 0.35
Molars	168	0.05 ± 0.04	0.07 ± 0.07	0.02 ± 0.02	0.55 ± 0.54	0.41 ± 0.34	0.18 ± 0.21
Total	649	0.05 ± 0.04	0.07 ± 0.06	0.02 ± 0.02	0.58 ± 0.50	0.49 ± 0.45	0.45 ± 0.48

^a^ Number of brackets used for calculation. ^b^ Absolute mean value ± standard deviation.

**Table 2:** Percentage of bracket positions outside the clinically acceptable transfer errors (±0.2 mm and ±1°) for version 1 (V1) and version 2 (V2) according to the direction of deviation.

**V1 ^b^**													
		**Mesiodistal (%)**		**Vertical (%)**		**Orovestibular (%)**		**Torque (%)**		**Rotation (%)**		**Tip (%)**	
	***n* ^a^**	**Mesial**	**Distal**	**Occlusal**	**Gingival**	**Oral**	**Vestibular**	**PCT**	**LCT**	**MR**	**DR**	**MCT**	**DCT**
Incisors	181	0.0	2.2	3.3	0.0	0.0	0.0	2.2	15.5	15.5	4.4	7.2	9.4
Canines	105	3.8	5.7	0.0	0.0	0.0	0.0	5.7	13.3	13.3	21.0	13.3	9.5
Premolars	198	2.0	0.0	0.5	1.5	0.0	0.0	8.1	7.1	7.1	11.6	3.5	4.0
Molars	198	1.0	0.0	0.5	3.6	0.0	0.0	10.8	1.5	1.5	5.2	1.0	0.5
Total	679	1.8	1.5	1.2	1.5	0.0	0.0	6.9	9.9	9.9	8.1	5.3	5.3
**V2 ^b^**													
		**Mesiodistal (%)**		**Vertical (%)**		**Orovestibular (%)**		**Torque (%)**		**Rotation (%)**		**Tip (%)**	
	***n* ^a^**	**Mesial**	**Distal**	**Occlusal**	**Gingival**	**Oral**	**Vestibular**	**PCT**	**LCT**	**MR**	**DR**	**MCT**	**DCT**
Incisors	186	0.0	0.5	1.6	1.6	0.5	0.0	11.8	3.2	9.1	3.2	11.8	3.2
Canines	101	1.0	2.0	1.0	2.0	0.0	0.0	18.8	3.0	11.9	5.9	12.9	5.9
Premolars	194	1.0	0.0	0.5	3.1	0.0	0.0	15.5	1.0	3.1	9.8	3.1	3.6
Molars	148	0.0	0.0	0.6	4.8	0.0	0.0	9.5	4.8	0.6	3.6	0.0	1.2
Total	649	0.3	0.2	0.9	2.9	0.2	0.0	13.4	2.9	5.5	5.7	7.4	4.6

^a^ Number of bonded teeth used for calculation. ^b^ Percentage of bracket positions outside the limit of 0.20 mm and 1°. PCT—palatal crown torque; LCT—labial crown torque; MR—mesiorotation; DR—distorotation; MCT—mesial crown tip; DCT—distal crown tip.

**Table 3:** A mixed model calculated the *p*-values to detect whether one factor or an interaction of two factors significantly influenced the positions of brackets in linear (mm values) and angular (degree axis) direction.

Factor(s)	*p*-Value (mm Values)	*p*-Value (Degree Values)
Versions ^a^	0.414	0.674
Versions * Tooth Groups ^b^	0.842	0.885

^a^ Version 1 and version 2. ^b^ Interaction effects (*) of two factors on axial and angular values.

## Data Availability

The data underlying this article will be shared on reasonable request to the corresponding authors.
